# Bibliography

**Published:** 1892-07

**Authors:** 


### Bibliography.

A Treatise on Dental Jurisprudence for Dentists and Lawyers. By William F. Rehfuss, D.D.S. Published by the Wilmington Dental Manufacturing Company. Philadelphia, 1892.

It may seem not only novel but somewhat out of place for a member of one profession to enter the domain of another and undertake a work of this character, and it may be doubted, if the case were reversed, whether a dentist would give a work on dentistry, prepared by a lawyer, a place in his library or even consider it worthy of notice. Yet it is not impossible for a lawyer to be theoretically an excellent dentist or even have a very thorough idea of general medicine, as both dentists and medical men have found to their chagrin, when called upon the witness stand. It is not unusual for one trained in the special work of one profession to have tastes which lead to study in the work of another. Ministers become lawyers and lawyers ministers, and both may become doctors of medicine. Examples are numerous of all these various changes.

The writer of the present volume has shown, through his persistent work of years, in compiling this volume of 468 pages, that, though an excellent dentist, his legal tastes have led him into a train of work, giving valuable results to his profession.

The idea will, doubtless, be generally entertained that, possibly, this book may not be prepared as a lawyer would arrange it, or, that it may be so defective in legal statement as to be practically valueless as a book of reference. This has been anticipated by the author, who has taken the precaution to have it revised by a well-known lawyer of Philadelphia, whose endorsement gives it all the professional strength required, and disarms criticism in this direction. He says, "I have examined the manuscript of your 'Dental Jurisprudence,' and find the general legal principles therein enunciated correct, while the arguments presented in the special subjects are in accordance with the authorized legal views. I can state that there are many facts in it which cannot fail to be of service to the advocate in the trial of a case, involving any part of the ground it covers.

"In the absence of any work on that particular subject, and especially from its intrinsic merit, your book is bound to become a reference in all cases of that kind."

The author explains his reasons for attempting this work in the preface, as follows: "Owing to the wonderful advancement within late years of the dental sciences, embracing the discovery of many new operations and methods of treatment, increased responsbilities are accredited to the dental surgeon, the neglect of which might involve him in litigation, and the knowledge thereof may at some period in his professional career avoid a calamity of a serious nature.

"For this reason a knowledge of dental jurisprudence would be of infinite value to the young graduate who too frequently enters upon his professional duties utterly ignorant and oblivious of the legal responsibilities incident to the practice of his profession."

The contents cover all possibilities that are likely to occur in practice, and become, therefore, of inestimable value to the beginner. The possibility of a suit at law for malpractice is a constant and increasing source of anxiety through all periods of the active life of a professional man, whether in dentistry or medicine. The very incorrect ideas entertained in regard to these various responsibilities has been often manifested in conventions whenever the matter has come up for discussion, and the writer has repeatedly heard it gravely stated that a graduate in dentistry was more liable to prosecution than the medically-educated man.

While there has been much written on medical jurisprudence, the question of the legal standing of the dentist has been almost entirely neglected, and with the exception of the able article on "Dental Jurisprudence," by Hon. Charles G. Garrison, in the "American System of Dentistry," there is nothing of special value. Hence, a book of this character is a timely production, coming as it does, at a period when the various States have enacted laws governing dental professional work.

The author divides his book under forty-two heads, among which may be found "The Legal Protection afforded the Dentist by the Degree of D.D.S." "Malpractice: What constitutes Dental Malpractice?" "Responsibilities of Dentists for one Error of Judgment." "Injuries and Deaths due to Anæsthesia, Compensations, etc."

A very full appendix is added, giving the laws of the several States and foreign countries. This is by no means the least valuable portion; indeed, it may be said to be very important, fulfilling, as it does, a want felt by all persons who have had occasion to refer to these enactments. The attempt has previously been made to bring these into an orderly, connected form, with only partial success. We have them here arranged for easy reference, and the collection will undoubtedly prove of great value to members of State boards, heads of colleges, newly-graduated students, and members of the legal profession.

While the compilation, as a whole, is made interesting by the citation of cases, the dentist will, perhaps, turn to pages which deal with his daily work and the legal possibilities connected therewith; and to show the mode of treatment of a subject, we give abstracts from that on "The Fracture of the Jaws."

"To uphold this charge it devolves upon the plaintiff to establish, to the satisfaction of the court, that such injury in reality exists, either by the affidavit or testimony of the physician called to treat the injury complained of, or it may involve the actual exhibition or examination of the injury in court. The plaintiff must also prove that the injury was received at the hands of the defendant unless it is admitted by the latter that he was the direct or indirect cause thereof.

"The next step in the proceedings is to demonstrate that the injury was due to malpractice, and to show the nature thereof; whether ignorant, due to want of skill, wilful or negligent malpractice, from carelessness in performing the operation."

The subject is then continued and considered in detail.

Under the head of "Authority and Legality of State Boards," the following quotation is not without interest. It is taken from a paper published by Judge McGary, of Washington, entitled, "Are Medical Laws Constitutional?" "The State may doubtless create a Board of Health or a similar body, and require every physician to register his license with the board, or in the office of the county clerk or other appropriate place. But his rights to practise will not depend upon his registration, as the right has been already vested and assured by the diploma of the college. And should the State also vest the Board of Health with the right and even the duty of examining and licensing applicants, it could not apply to those who held a license from the incorporated colleges. Nor could it militate against or invalidate in any way the right of the colleges to issue valid diplomas and licenses before or after; for not even can the State, by its legislation, divest or impair a vested right which the colleges already had."

The question of "Patent Rights" occupies, as it naturally would, considerable space, and very clearly states the law in the question, together with a detailed account of the many prominent cases that have disturbed the dental profession in the past and are a source of anxiety in the present.

Perhaps that portion of the book already alluded to, "Dental Laws in Foreign Countries," will be found most acceptable to a certain class of inquirers. The difficulty of acquiring exact information in regard to these has been a source of much embarrassment to deans of colleges, as they, more than others, are obliged to keep themselves informed in regard thereto; but the difficulties in arriving at this information have heretofore been almost insurmountable. The author has, therefore, performed a most excellent service in adding this part to his work. It covers the laws of Austria, Hungary, Belgium, Brazil, Cuba, Denmark, France, Germany, Great Britain, New Zealand, Dominion of Canada and Provinces, Holland, Italy, Russia, and Spain.

While the critical lawyer may find some things to take exception to, the non-legal mind will, we think, regard the work as most satisfactory. The careful reading of its well-prepared pages will unquestionably be of value to many who have failed to consider the serious side of professional work.

The author is to be congratulated that in this new field of labor he has been able to add something of positive value to the needs of his profession.

The book is produced with excellent taste, and the publishers—The Wilmington Dental Manufacturing Company—deserve more than ordinary credit for their courage in assuming a business responsibility which other firms had previously declined. The dental profession should show by the demand for the book that their conclusions were not misplaced, and aid in giving it the widest circulation.

